# Thai Medicinal Flowers as Natural Antioxidants and Antibacterial Agents Against Pathogenic Enteric Bacteria: A Comparative Study of *Mesua ferrea*, *Mammea siamensis*, and *Clitoria ternatea*

**DOI:** 10.3390/antibiotics14101038

**Published:** 2025-10-16

**Authors:** Sureeporn Suriyaprom, Nitsanat Cheepchirasuk, Pornpimon Ngamsaard, Varachaya Intachaisri, Angkhana Inta, Yingmanee Tragoolpua

**Affiliations:** 1 Office of Research Administration, Chiang Mai University, Chiang Mai 50200, Thailand; sureeporn.suriyaprom@cmu.ac.th (S.S.); varachaya.int@cmu.ac.th (V.I.); 2 Department of Biology, Faculty of Science, Chiang Mai University, Chiang Mai 50200, Thailand; nitsanat_cheep@cmu.ac.th (N.C.); promjinnapimon@gmail.com (P.N.); angkhana.inta@cmu.ac.th (A.I.); 3 Doctor of Philosophy Program in Applied Microbiology (International Program), Faculty of Science, Chiang Mai University, Chiang Mai 50200, Thailand; 4 Natural Extracts and Innovative Products for Alternative Healthcare Research Group, Faculty of Science, Chiang Mai University, Chiang Mai 50200, Thailand

**Keywords:** Thai medicinal flowers, antibacterial, antioxidant, biofilm, bacterial adhesion, enteric pathogens

## Abstract

Thai medicinal flowers, namely *Mesua ferrea* L. (Bunnak), *Mammea siamensis* T. Anderson (Saraphi), and *Clitoria ternatea* (Anchan) have long been valued for their traditional medicinal. This study investigated their phytochemical composition and bioactivities, with a particular focus on antioxidant and antibacterial properties. Methods: Ethanolic flower extracts were analyzed by high-performance liquid chromatography (HPLC) and liquid chromatography–mass spectrometry (LC–MS). Antioxidant activities were determined by DPPH, ABTS, and FRAP assays. Antibacterial activity against *Escherichia coli*, *E. coli* O157:H7, *Salmonella* Typhi, *Shigella dysenteriae*, and *Vibrio cholerae* were assessed by agar well diffusion, broth dilution methods, and time–kill assays. Biofilm formation, biofilm disruption, and bacterial adhesion to Caco-2 cells were evaluated. Morphological changes in *E. coli* O157:H7 were examined using scanning electron microscopy (SEM), and leakage of intracellular contents (DNA, RNA, proteins) were quantified. Results: HPLC analysis revealed the highest level of gallic acid in *M. ferrea* and quercetin in *M. siamensis*. LC–MS analysis identified fifteen putative metabolites across the flower extracts, including quercetin, kaempferol, catechin, and luteolin derivatives, with species-specific profiles. *C. ternatea* extract exhibited the greatest total flavonoid content and antioxidant activity. Among the extracts, *M. ferrea* exhibited the strongest inhibitory effect, with inhibition zone of 13.00–15.00 mm and MIC/MBC values of 31.25–62.5 mg/mL. All extracts exhibited time-dependent bactericidal activity, significantly inhibited biofilm formation, disrupted established biofilms, and reduced bacterial adhesion to intestinal epithelial cells. SEM revealed membrane disruption in *E. coli* O157:H7 and leakage of intracellular components. Conclusions: Thai medicinal flower extracts, particularly *M. ferrea*, possess strong antioxidant and antibacterial activities. Their ability to inhibit biofilm formation, interfere with bacterial adhesion, and disrupt bacterial membranes highlights their potential as natural alternatives for preventing or controlling enteric bacterial infections.

## 1. Introduction

Enteric bacterial pathogens are major contributors to foodborne gastroenteritis in humans, representing a significant global public health issue [1]. Notable pathogens in this category include *Salmonella* spp., *Escherichia coli*, *Listeria monocytogenes*, *Vibrio cholerae*, *Norovirus*, and *Campylobacter* [2]. The widespread and often inappropriate use of antibiotics in humans, animals, and agriculture has exacerbated the prevalence of infectious diseases and accelerated the emergence of antimicrobial resistance, particularly in developing countries [1]. This growing burden of drug-resistant infections, caused by both pathogenic and opportunistic microorganisms, presents a serious threat to public health and poses an urgent challenge to healthcare systems worldwide.

In response to the challenges posed by antimicrobial resistance, there has been growing interest in natural antimicrobial agents as alternatives to synthetic chemicals. These natural agents are often perceived by consumers as safer and more environmentally friendly. Among natural sources, flowering plants and their extracts, widely used to enhance the aroma, flavor, and color of food, have gained considerable attention for their potential as natural food preservatives [3]. Numerous studies have highlighted the bioactive properties of these plant-derived substances, particularly their antimicrobial activities, alongside other health-promoting benefits [4]. Herbal plants have long been utilized in folk medicine across various cultures and offer a diverse range of health benefits. Natural extracts from different parts of medicinal plants, such as fruits, seeds, leaves, bark, flowers, and roots, are recognized for their potential applications in nutritional enhancement, food preservation, and therapeutic use [5]. These plant-based compounds often exhibit a broad spectrum of pharmacological activities, including antimicrobial, anti-inflammatory, antioxidant, and anticancer effects. These effects made them invaluable for developing functional foods and alternative medicines [5,6]. In Southeast Asia, flowering medicinal plants have long held a central place in traditional healing practices. Among these, three Thai native species, *Mesua ferrea* Linn. (Bunnak), *Mammea siamensis* T. Anderson (Saraphi), and *Clitoria ternatea* L. (Anchan or butterfly pea), are particularly notable for their historical use in folk remedies and their promising pharmacological properties.

*Mesua ferrea* Linn., commonly known as Bunnak, is a perennial evergreen tree in the Clusiaceae family, found predominantly in Thailand, Malaysia, India, Sri Lanka, and parts of southern China such as Yunnan and Guangxi. Both flowers and leaves are traditionally used for medicinal purposes [7]. Phytochemical analyses have identified a range of bioactive compounds in the flowers, including coumarins, flavonoids, triterpenoids, and mangiferic acids [8]. Modern pharmacological studies support its antibacterial, anti-inflammatory, anticholinesterase, antioxidant, and anti-platelet aggregation effects [7], highlighting its potential for development into therapeutic agents and functional foods. Another important species, *Mammea siamensis* T. Anderson (Saraphi), is a small evergreen tree from the Calophyllaceae family, native to Thailand and neighboring countries such as Laos, Cambodia, Vietnam, and Myanmar. Its fragrant yellow or white blossoms have been used in Thai traditional medicine as a heart tonic, fever reducer, and appetite stimulant [9,10]. *Clitoria ternatea* L., widely known as butterfly pea or Anchan, is a climbing plant from the Fabaceae family commonly found throughout tropical and subtropical regions. Its roots, flowers, leaves, and seeds are traditionally used in Indian and Southeast Asian folk medicine and recognized for their diverse therapeutic properties [11]. The vivid blue flowers are particularly rich in flavonols, such as quercetin, myricetin, and kaempferol derivatives. Anthocyanins including various ternatin compounds (A1–A3, B1–B4, C1–C4, and D1–D3) have also been identified in the flowers. These compounds contribute to wide applications of the flowers in traditional remedies, cosmetics, and as a natural food colorant in culinary practices [12].

To explore the potential applications of these flowers, their extracts were analyzed for bioactive components, including total phenolics and flavonoids, and the evaluation of antioxidant capacity. Quantitative measurements of gallic acid and quercetin were carried out using high-performance liquid chromatography (HPLC), while liquid chromatography–mass spectrometry (LC–MS) was employed to identify and profile a broader range of phenolic and flavonoid metabolites. The antibacterial activity of these flowers was examined against pathogenic enteric bacteria, emphasizing the inhibition of bacterial growth, suppression of biofilm formation, and reduction in bacterial adhesion to intestinal epithelial cells. Furthermore, structural changes in Escherichia *coli* O157:H7 and the release of intracellular substances, such as DNA, RNA, and proteins, were investigated to clarify the underlying antibacterial mechanisms.

## 2. Results

### 2.1. Flower Extraction

Dried flowers of *M. ferrea*, *M. siamensis*, and *C. ternatea* were extracted using 95% ethanol. The percentage yields of the flower extracts are presented in Table 1. Among the three, the *M. ferrea* extract exhibited the highest yield at 18.91%, followed by *C. ternatea* (17.51%) and *M. siamensis* (16.03%).

### 2.2. Phytochemical Compounds in Flower Extracts

**Figure 1:**
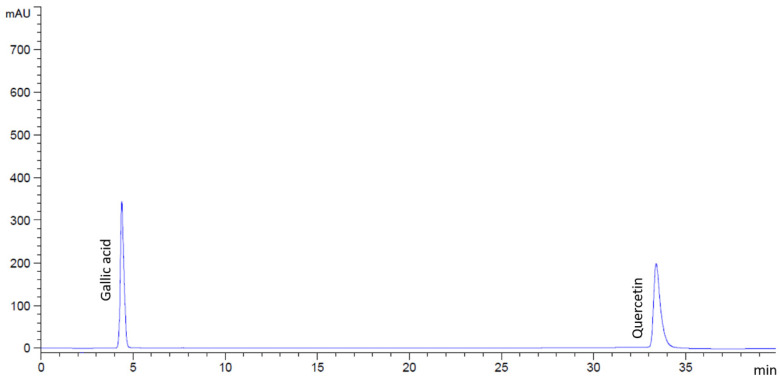
HPLC chromatograms of the standard mixture containing gallic acid and quercetin.

**Figure 2:**
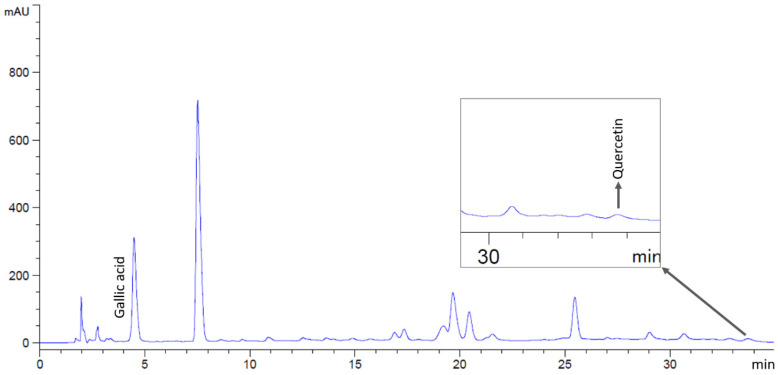
HPLC chromatograms of *M. ferrea* extract, indicating peaks corresponding to gallic acid and quercetin.

**Figure 3:**
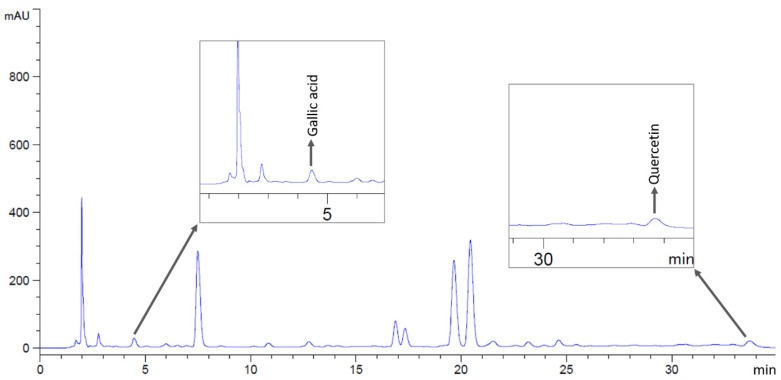
HPLC chromatograms of *M. siamensis* extract, indicating peaks corresponding to gallic acid and quercetin.

**Figure 4:**
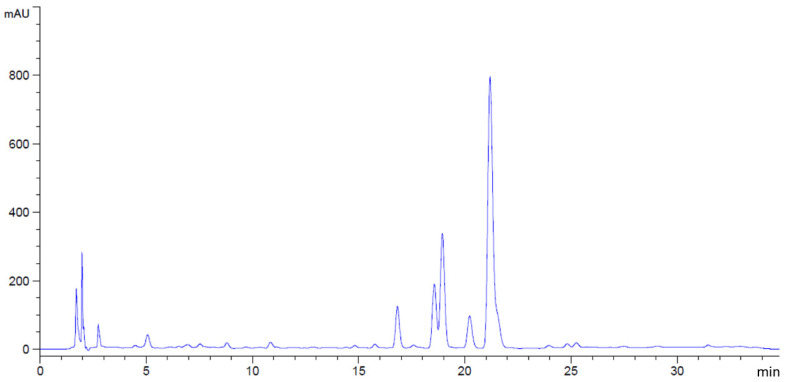
HPLC chromatograms of *C. ternatea* extract. Gallic acid and quercetin were not detected in this study.

Phytochemical compounds in the flower extracts were quantified using high-performance liquid chromatography (HPLC). The levels of gallic acid and quercetin in *M. ferrea*, *M. siamensis*, and *C. ternatea* extracts were analyzed in this study (Figure 1). The *M. ferrea* extract contained the highest amount of gallic acid (16.956 ± 0.059 mg/g extract), followed by *M. siamensis* (0.921 ± 0.015 mg/g extract). Similarly, the quercetin content was significantly higher in the *M. siamensis* extract (0.678 ± 0.025 mg/g extract), followed by *M. ferrea* (0.260 ± 0.027 mg/g extract) (Table 2 and Figure 2 and Figure 3). In contrast, neither gallic acid nor quercetin was detected in the *C. ternatea* extract under the HPLC conditions used (Figure 4). Nevertheless, comprehensive chemical profiles of other bioactive compounds in the flower extracts were also demonstrated using LC–MS analysis.

**Figure 5:**
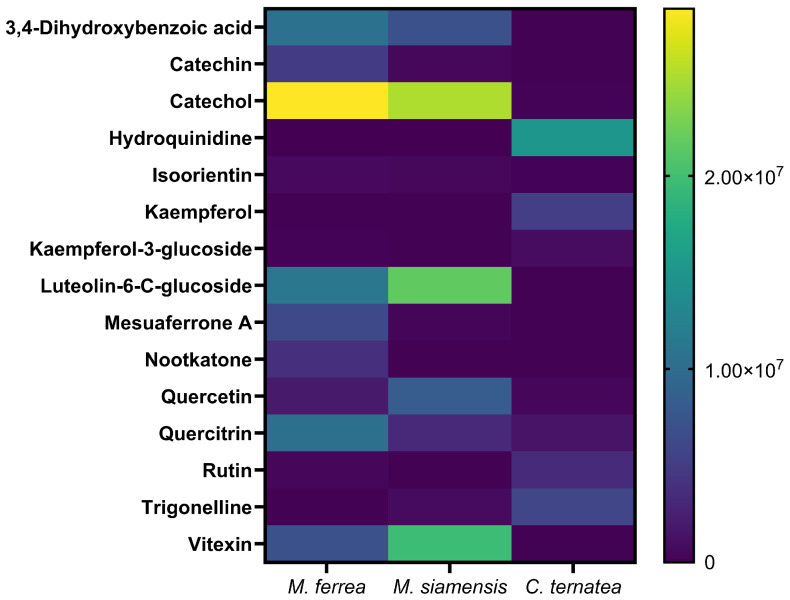
Heat map of LC–MS–identified metabolites in flower extracts.

To further verify and expand the chemical profiling results, liquid chromatography–mass spectrometry (LC–MS) analysis was performed. The total ion chromatograms revealed a diverse array of metabolites eluting at different retention times across the three flower extracts. A total of fifteen putative metabolites were identified with high confidence (identification scores ≥ 1.50), as summarized in Figure 5. Among these, kaempferol exhibited the highest identification score (1.73), indicating strong reliability in compound assignment. Other detected major phenolic compounds included catechol, 3,4-dihydroxybenzoic acid, catechin, quercetin, rutin, and luteolin-6-C-glucoside. Interestingly, LC–MS analysis also confirmed the presence of HPLC-quantified compounds, namely gallic acid and quercetin, in *M. ferrea* and *M. siamensis*, while their signals were negligible in *C. ternatea* (Table S1). Additionally, species-specific metabolites were observed. Mesuaferrone A and nootkatone were abundant in *M. ferrea* whereas trigonelline and kaempferol derivatives predominated in *C. ternatea*.

### 2.3. Total Phenolic and Flavonoid Contents in Flower Extracts

The bioactive compounds in the flower extracts were identified by determining their total phenolic and flavonoid contents, as presented in Table 3. The *M. ferrea* extract exhibited the highest total phenolic content (50.09 ± 1.01 milligram gallic acid equivalents per gram of extract; mg GAE/g extract), followed by *M. siamensis* (26.02 ± 0.62 mg GAE/g extract) and *C. ternatea* (10.66 ± 0.85 mg GAE/g extract). In contrast, the highest total flavonoid content was observed in the *C. ternatea* extract (19.37 ± 0.91 milligram quercetin equivalent per gram of extract; mg QE/g extract), followed by *M. siamensis* (16.51 ± 0.01 mg QE/g extract) and *M. ferrea* (12.48 ± 0.48 mg QE/g extract). All differences were statistically significant.

### 2.4. Antioxidant Activities of Flower Extracts

Antioxidant properties of the flower extracts were determined using DPPH, ABTS, and FRAP assays. IC_50_ values, which indicate the concentration of extract that needed to neutralize 50% of free radicals, were calculated for both DPPH and ABTS assays. Among all tested extracts, *C. ternatea* showed the strongest antioxidant activity, with IC_50_ values of 0.30 mg/mL (DPPH) and 1.00 mg/mL (ABTS) (Table 4). The activities were also compared to reference standards, gallic acid for DPPH and Trolox for ABTS. In these comparisons, *C. ternatea* extract presented the highest antioxidant capacity, with 16.48 mg GAE/g extract in the DPPH assay and 159.46 mg TEAC/g extract in the ABTS assay. In contrast, the highest FRAP value was observed in *M. ferrea* extract, equivalent to 186.49 mg FeSO_4_/g extract.

### 2.5. Antibacterial Activities of Flower Extracts

**Figure 6:**
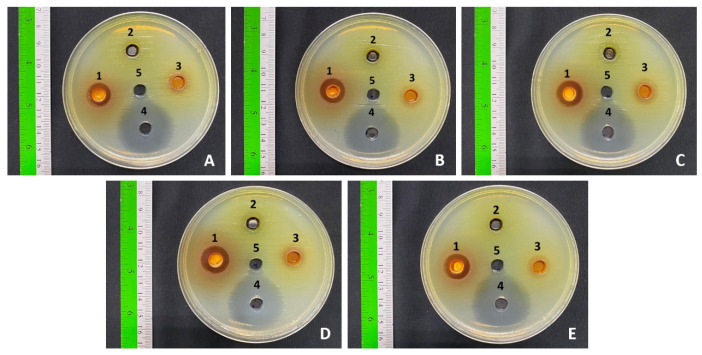
Antibacterial activity of (1) *M. ferrea* extract, (2) *C. ternatea* extract, (3) *M. siamensis* extract, (4) gentamicin (1 mg/mL), and (5) DMSO (99.9%) against pathogenic enteric bacteria: (**A**) *E. coli*, (**B**) *E. coli* O157:H7, (**C**) *S.* Typhi, (**D**) *S. dysenteriae*, and (**E**) *V. cholerae*.

The antibacterial potential of the flower extracts (500 mg/mL) was examined against major foodborne and enteric pathogens, including *E. coli*, *E. coli* O157:H7, *S.* Typhi DMST 22842, *S. dysenteriae* DMST 1511, and *V. cholerae*, using the agar well diffusion assay. Inhibition zone diameters were recorded to evaluate the effectiveness of each extract. Among the tested samples, *M. ferrea* extract demonstrated strong antibacterial activity, with inhibition zones ranging from 13.00 to 15.00 mm (Table 5 and Figure 6). The *M. siamensis* extract inhibited *E. coli*, *S.* Typhi, and *S. dysenteriae*, with inhibition zones ranging from 10.00 to 11.00 mm. In contrast, *C. ternatea* extract showed no inhibitory effect against any of the tested bacteria. The positive control (gentamicin at 1 mg/mL) produced inhibition zones of 26.67–29.33 mm, while no clear zones were observed in the negative control (99.9% DMSO).

The antibacterial potency of the flower extracts was assessed by determining the minimum inhibitory concentration (MIC) and minimum bactericidal concentration (MBC) using the broth dilution technique. All tested bacterial species were susceptible to the extracts, with MIC and MBC values ranging between 31.25 and 125 mg/mL (Table 6). The *M. ferrea* extract exhibited the highest activity against *S.* Typhi, *S. dysenteriae*, and *V. cholerae*, with both MIC and MBC values of 31.25 mg/mL. In contrast, the *C. ternatea* extract showed the lowest activity against all tested bacteria, with MIC and MBC values of 125 mg/mL. Gentamicin, used as a positive control, effectively inhibited the growth of all tested bacteria, with MIC and MBC values of 0.0078 mg/mL.

**Figure 7:**
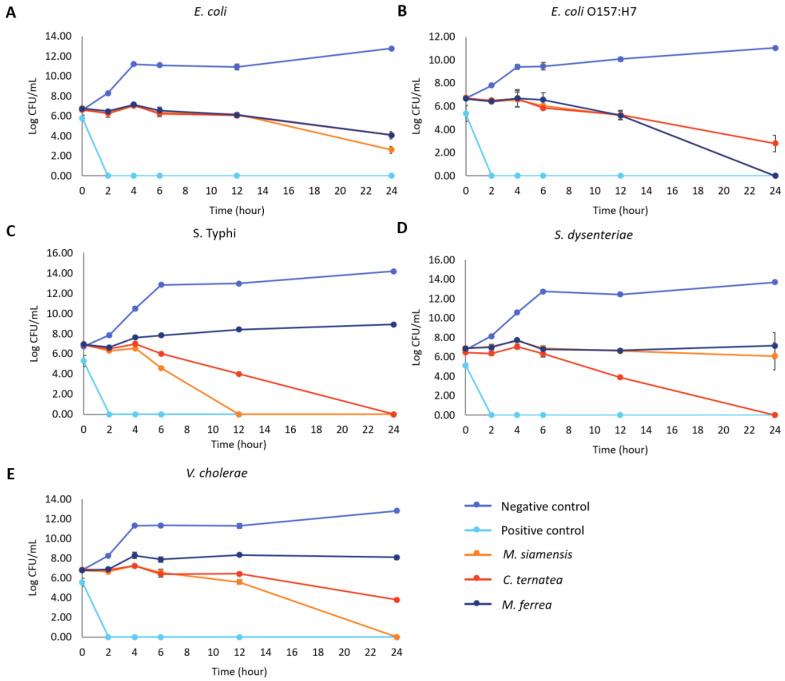
Effect of flower extracts (at 1 MIC) against (**A**) *E. coli*, (**B**) *E. coli* O157:H7, (**C**) *S.* Typhi, (**D**) *S. dysenteriae*, and (**E**) *V. cholerae*. Time–kill data are presented as mean ± SD (*n* = 3). Gentamicin at 1 mg/mL was used as the positive control, and untreated bacteria served as the negative control.

The bactericidal efficacy of the flower extracts was assessed by monitoring the time required to eradicate the tested bacteria. The time to achieve no viable cell count varied among the bacterial species and flower extracts (Figure 7). *M. ferrea* extract completely eradicated *E. coli* O157:H7 within 24 h, while *M. siamensis* extract completely inhibited *S.* Typhi within 12 h. *C. ternatea* extract achieved the same result for *S.* Typhi within 24 h. Furthermore, *C. ternatea* extract completely inhibited *S. dysenteriae* after 24 h, and *M. siamensis* extract completely killed *V. cholerae* within 24 h. The flower extracts exhibited the slowest killing rate against *E. coli*, taking 24 h to inhibit more than 60% of the bacteria (Table S2).

### 2.6. Antibiofilm Activity of Flower Extracts

**Figure 8:**
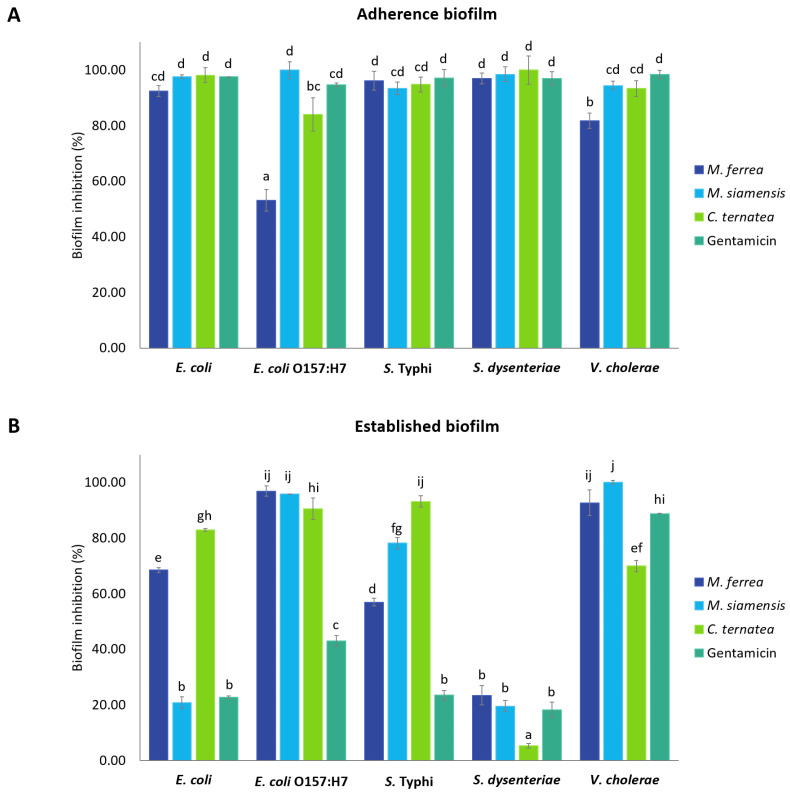
Inhibition of (**A**) adherence biofilm and (**B**) established biofilm by flower extracts against selected pathogenic enteric bacteria. Data are presented as mean ± standard deviation (*n* = 3). Statistical significance was indicated by different letters (a–j), representing significant differences (*p* < 0.05). Statistical analysis was performed using one-way ANOVA followed by Tukey’s post hoc test.

The antibiofilm activity of the flower extracts was evaluated using the crystal violet assay to assess their potential to inhibit biofilm formation. The inhibitory effects on both bacterial adherence during biofilm development and on pre-formed (established) biofilms are illustrated in Figure 8 and Tables S3 and S4. The extracts were tested at their respective MIC values, and the results are expressed as percentage inhibition relative to the untreated control group. Inhibition of bacterial adherence during biofilm formation ranged from 53.18% to 100% (Figure 8A). *M. siamensis* and *C. ternatea* extracts at their MIC values showed the highest efficacy against *E. coli*, *S. dysenteriae*, and *V. cholerae*, with inhibition rates between 93.38% and 100%. Similarly, *M. ferrea* extract effectively inhibited biofilm formation in *S.* Typhi, with inhibition rates of 96.20%. In contrast, it showed the lowest inhibitory effect on *E. coli* O157:H7, with inhibition rates of approximately 53.18%.

Regarding the inhibition of established biofilms, the flower extracts exhibited inhibition rates ranging from 5.25% to 100% (Figure 8B). *M. siamensis* extract demonstrated the highest inhibitory activity against established biofilms formed by *E. coli* O157:H7, with inhibition rates of 95.90%, and showed complete inhibition (100%) of *V. cholerae* biofilm formation. Additionally, *M. ferrea* extract exhibited significant activity against *E. coli* O157:H7 biofilm formation, with an inhibition rate of 96.90%. Although *C. ternatea* extract showed a strong inhibitory effect against *E. coli* (82.90%) and *S.* Typhi (93.14%) biofilm formation, it exhibited the lowest activity against *S. dysenteriae*, with an inhibition rate of only 5.25%. Gentamicin, used as the positive control, inhibited bacterial adherence during biofilm formation and against established biofilms, with inhibition rates ranging from 94.71% to 98.43% and 18.21% to 88.81%, respectively.

### 2.7. Antibacterial Adhesion Activity of Flower Extracts on Caco-2 Cells

The ability of the flower extracts to inhibit bacterial adhesion was assessed using Caco-2 cells. Before conducting the adhesion assay, cytotoxicity of the extracts toward Caco-2 cells was examined by MTT assay (Figure S1 and Table S5). A vehicle control (1% DMSO) was also included, no significant difference in cell viability was observed compared with the untreated control, indicating that DMSO at this concentration had no cytotoxic effect. At concentrations of 0.08 mg/mL (*M. ferrea*), 0.0024 mg/mL (*M. siamensis*), and 0.63 mg/mL (*C. ternatea*), cell viability remained comparable to the untreated control. The IC_50_ values and the corresponding selectivity indices (SI), calculated as the ratio of IC_50_ to the minimum inhibitory concentration (MIC), are presented in Table S6. These data quantitatively confirm the safety of the extracts, demonstrating that the concentrations used in the adhesion assay were well below the cytotoxic threshold.

**Figure 9:**
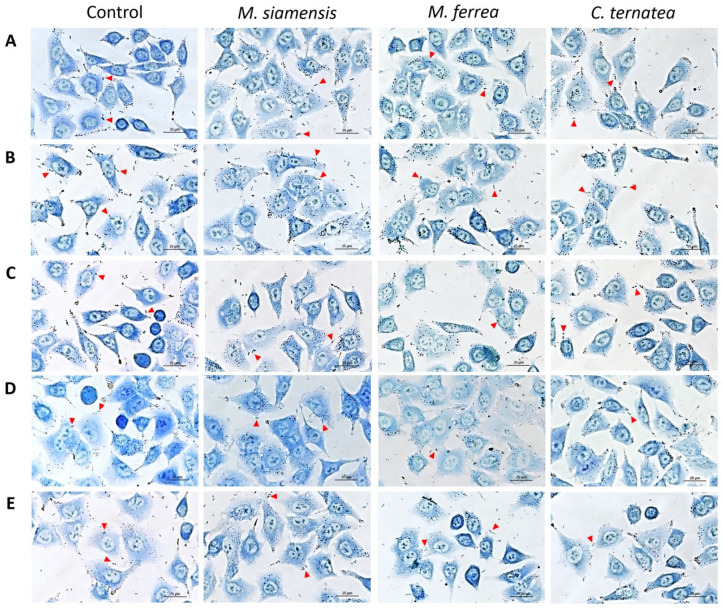
Effect of flower extracts on bacterial adhesion to Caco-2 cells. Adhesion of (**A**) *E. coli*, (**B**) *E. coli* O157:H7, (**C**) *S.* Typhi, (**D**) *S. dysenteriae*, and (**E**) *V. cholerae* to Caco-2 cells following treatment with flower extracts. Cells were stained with 0.1% methylene blue and examined under a light microscope. The red triangles indicate representative adhered bacterial cells. Images were captured at 400× magnification.

The antibacterial adhesion activity of the flower extracts was expressed as a percentage relative to the untreated control. Inhibition of bacterial adhesion ranged from 57.41% to 83.14%, as shown in Table 7 and Figure 9. Among the tested extracts, *M. siamensis* and *C. ternatea* demonstrated the highest activity against the adhesion of *S.* Typhi to Caco-2 cells, with inhibition rates of 57.41% and 55.18%, respectively. *M. ferrea* exhibited inhibitory activity against *Escherichia coli* (20.49%); however, it showed the lowest efficacy in reducing *S.* Typhi adhesion, with only 3.14% inhibition. *M. siamensis* demonstrated the highest inhibitory effect on *V. cholerae* adhesion, while *C. ternatea* was most effective against *E. coli* O157:H7. In contrast, all flower extracts exhibited relatively low inhibitory activity against the adhesion of *S. dysenteriae*.

### 2.8. Effects of Flower Extracts on the Cellular Structure of E. coli O157:H7

**Figure 10:**
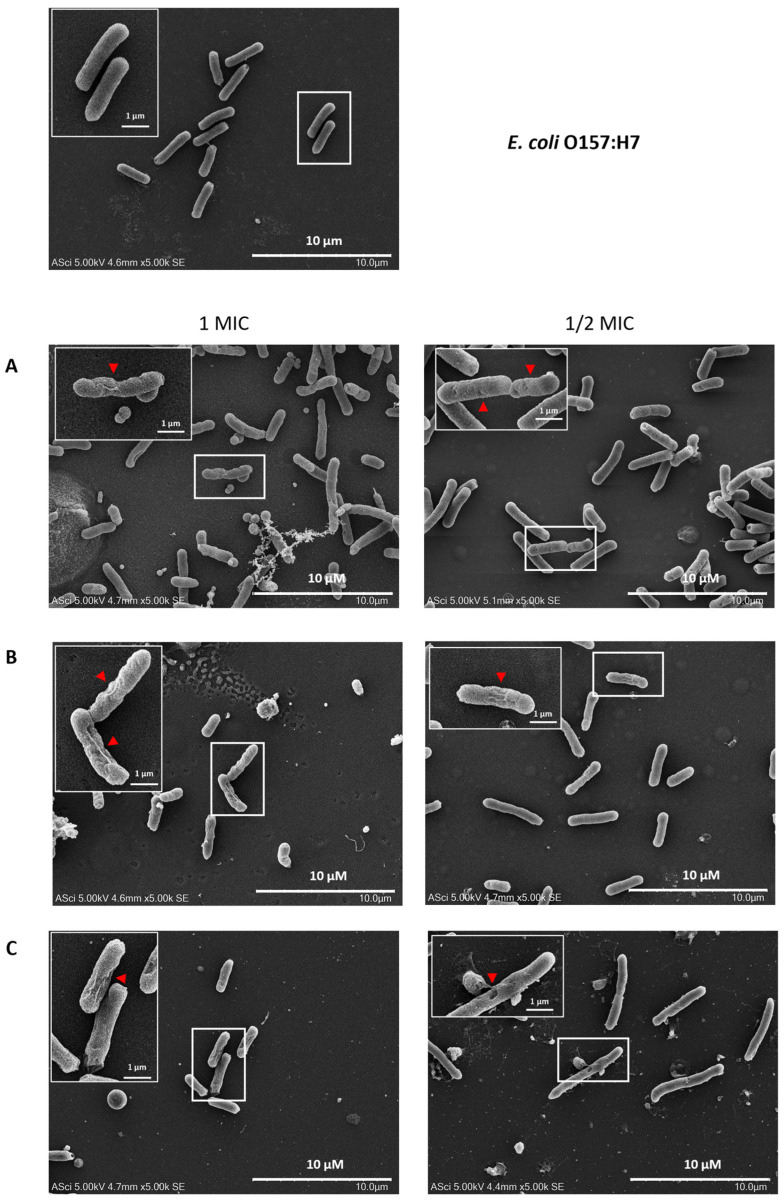
Morphological changes in *E. coli* O157:H7 following treatment with (**A**) *M. ferrea*, (**B**) *M. siamensis*, and (**C**) *C. ternatea*, as observed under a scanning electron microscope. The red triangles indicate representative distorted and damaged bacterial cells.

The morphology of *E. coli* O157:H7 treated with flower extracts at 1 and ½ MIC was examined using scanning electron microscopy (SEM), revealing marked morphological alterations. As shown in Figure 10, untreated *E. coli* O157:H7 cells exhibited a normal, rod-shaped structure with smooth and intact surfaces. In contrast, cells treated with flower extracts displayed significant morphological disruptions, including distorted shapes, wrinkled and damaged surfaces, and irregular, shriveled, and rugged appearances. These morphological changes suggest that the flower extracts induce structural damage to the bacterial cell envelope, indicating their potential antibacterial mechanism of action.

**Figure 11:**
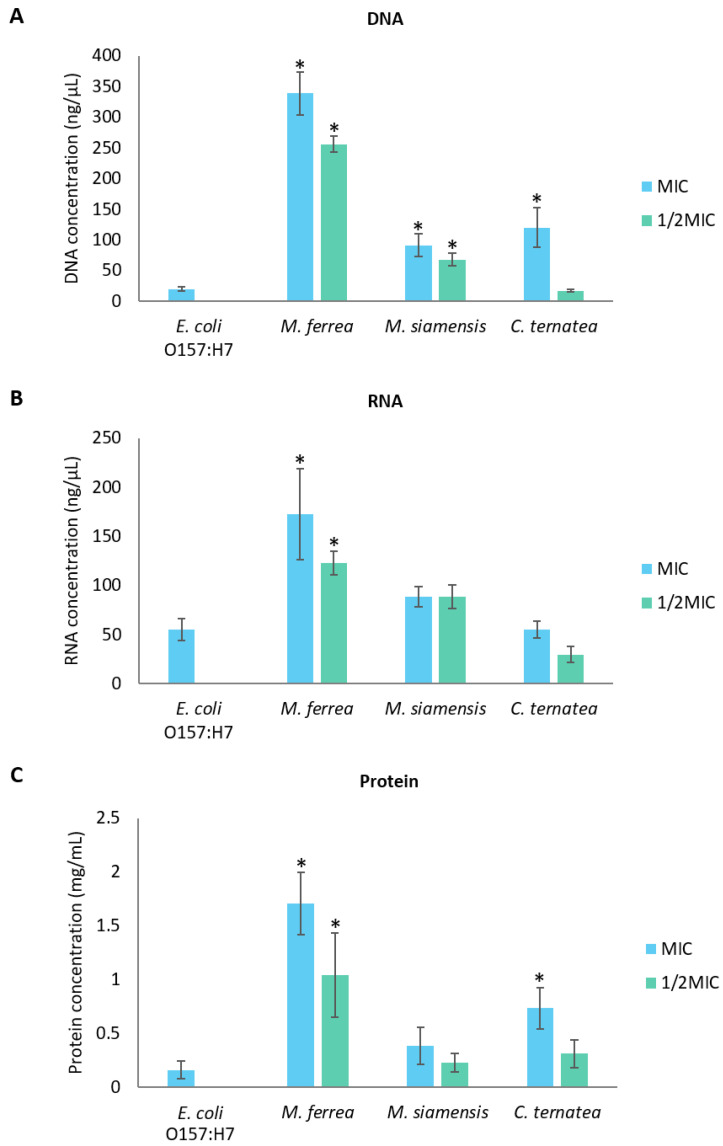
Effects of flower extracts on the leakage of (**A**) DNA, (**B**) RNA, and (**C**) protein from *E. coli* O157:H7. * Indicates significant difference (*p* < 0.05) compared with the untreated *E. coli* O157:H7 control group.

The influence of flower extracts on the integrity of bacterial cell membranes was investigated by measuring the release of DNA, RNA, and proteins from *E. coli* O157:H7 into the extracellular medium. As shown in Figure 11, the levels of extracellular DNA and RNA increased with increasing concentrations of the flower extracts. A similar trend was observed for protein leakage (Figure 11C). These results indicate that the leakage of DNA, RNA, and protein was concentration-dependent, with higher concentrations of the extracts causing more pronounced effects.

## 3. Discussion

Several phytochemical studies have demonstrated that Thai flowers such as *Mesua ferrea*, *Mammea siamensis*, and *Clitoria ternatea* contain a diverse array of bioactive constituents, including flavonoids, phenolic acids, and other polyphenolic compounds. These phytochemicals are known to contribute to the antioxidant, antibacterial, and anti-inflammatory properties, supporting their traditional medicinal uses and highlighting their therapeutic potential. In this study, the HPLC analysis revealed varying levels of gallic acid and quercetin among the flower extracts, which were selected as representative markers of phenolic acids and flavonoids, respectively. These two compounds were chosen as standards because they are well-characterized and commonly used as indicators of total phenolic and flavonoid content, allowing for reliable comparison across samples [13]. Although gallic acid and quercetin do not fully capture the phytochemical complexity of the extracts, they provide suitable indicators for linking chemical composition with the observed antioxidant and antibacterial activities [14]. *M. ferrea* exhibited the highest gallic acid content (16.956 ± 0.059 mg/g extract), which aligns with previous findings reporting gallic acid and quercetin levels of 13.17 mg/g extract and 0.34 mg/g extract, respectively [15]. Additionally, other phenolic compounds such as coumaric acid, catechin, rutin, and ferulic acid have also been identified in *M. ferrea* ethanolic extracts, highlighting its rich polyphenolic profile. In contrast, gallic acid and quercetin were not detected in the *C. ternatea* extract, which differs from earlier reports identifying these compounds in the same species [16]. However, our findings are consistent with those of Escher et al. (2020), who also failed to detect quercetin using HPLC-PAD-UV [11]. Interestingly, despite the non-detection of gallic acid and quercetin, *C. ternatea* extract exhibited the highest total flavonoid content and demonstrated strong antioxidant activity in both DPPH and ABTS assays. Previous studies have primarily focused on the antioxidant activity of *C. ternatea*, with reported IC_50_ values in the DPPH assay ranging from 0.08 to 4 mg/mL [12], further supporting its potent antioxidant capacity. The discrepancy between the absence of detectable gallic acid and quercetin and the observed antioxidant activity may be explained by the presence of other flavonoid compounds that were either not specifically targeted or present at concentrations below the detection limit of HPLC [17]. Moreover, total flavonoid and antioxidant assays detect a broader range of phenolic and flavonoid structures, many of which may not be individually identified by standard HPLC methods [18]. The discrepancy between the HPLC profile and antioxidant activity suggests that other bioactive flavonoids may contribute to the functional properties of the extract.

To further explore this, LC–MS analysis was conducted to obtain a more comprehensive chemical profile of the extracts. The LC–MS results confirmed the chemical diversity among the three species and revealed several metabolites. Specifically, in *C. ternatea*, compounds such as kaempferol derivatives, trigonelline, and hydroquinidine were identified, which have been reported to possess both antioxidant and antibacterial properties [19,20,21,22]. These results are consistent with *C. ternatea* extract exhibiting the highest total flavonoid content and strongest antioxidant activity. The additional peaks observed in the HPLC chromatogram of *C. ternatea*, particularly between 15 and 25 min, may correspond to these flavonoid and phenolic compounds detected by LC–MS. Therefore, these findings suggest that the biological activities of *C. ternatea* are attributed to the synergistic effects of diverse flavonoid and phenolic constituents, rather than the presence of gallic acid or quercetin alone.

Regarding *M. ferrea*, the extract exhibited the highest total phenolic content among the tested samples. HPLC analysis identified *p*-coumaric acid and gallic acid as the predominant phenolic acids, while rutin was the major flavonoid detected in the extract [15]. The elevated total phenolic content may be largely attributed to the abundant presence of gallic acid and quercetin, both of which are well-documented for their potent antioxidant properties [23]. This is consistent with the FRAP assay results, where *M. ferrea* extract demonstrated the highest antioxidant activity, further supporting the role of these phenolic compounds in contributing to its antioxidant potential. Several studies have shown that *p*-coumaric acid, gallic acid, and ferulic acid exhibit strong antioxidant activity across various assays, including DPPH, ABTS, FRAP, and ORAC [24]. Collectively, these compounds enhance the free radical scavenging ability of the extract, supporting the observed antioxidant effects.

Among the tested flower extracts, *M. ferrea* demonstrated the most potent antibacterial activity, effectively inhibiting all tested pathogenic enteric bacteria, including *E. coli*, *E. coli* O157:H7, *S.* Typhi, *S. dysenteriae*, and *V. cholerae*. In comparison, *M. siamensis* extract showed inhibitory activity against *E. coli*, *S.* Typhi, and *S. dysenteriae*, while *C. ternatea* extract exhibited no inhibitory effect against any of the tested bacteria under the same experimental conditions using the agar well diffusion method. Nevertheless, all tested extracts demonstrated bactericidal activity in broth microdilution assays, with MIC and MBC values being equal, ranging from 31.25 to 125 mg/mL. These findings contrast with a previous study, which reported that ethanolic extracts of *M. ferrea* and *M. siamensis* at 500 mg/mL inhibited *Staphylococcus aureus*, but not *E. coli*, *S. dysenteriae*, or *S.* Typhimurium when tested using the agar disk diffusion method [25].

Although *C. ternatea* extract exhibited no antibacterial activity against enteric pathogens in the agar well diffusion assay, it demonstrated clear inhibitory effects in the broth microdilution assay, as indicated by measurable MIC and MBC values. This discrepancy suggests that its antibacterial efficacy may be method-dependent, potentially influenced by factors such as compound diffusion efficiency and solubility. These limitations are well-documented in agar-based methods, where compounds with low diffusion capacity may not produce observable inhibition zones despite possessing antimicrobial activity [26]. Since agar diffusion assays rely heavily on the ability of compounds to permeate through the agar matrix, substances with high molecular weight, low aqueous solubility, or lipophilic characteristics may fail to diffuse adequately, thus underestimating their true antibacterial potential [27]. In contrast, the broth microdilution method enables uniform distribution of test substances in a liquid medium, ensuring consistent exposure between bacteria and the compounds throughout incubation [28]. This allows for more accurate detection of inhibitory activity, particularly in the case of slow-acting or low-potency antimicrobials, and offers greater sensitivity and quantification capacity compared to diffusion-based methods [29]. These findings emphasize the importance of employing complementary methodologies when evaluating the antibacterial potential of plant-derived compounds and highlight the limitations of relying solely on agar-based techniques.

Moreover, time–kill assays further substantiated the antibacterial efficacy of the flower extracts by enabling real-time monitoring of bacterial growth [29]. The extracts exhibited time-dependent bactericidal activity, as evidenced by extended lag phases and slowed log phases in all tested enteric pathogens. The prolonged lag phase reflects the bacterial adaptation period following exposure to antimicrobial stress, during which macromolecular damage may be repaired and essential cellular components synthesized for growth resumption [4]. This suggests that the flower extracts may interfere with key biosynthetic processes involved in macromolecule production. Additionally, the varied responses among bacterial species may be attributed to differences in the composition and structure of their lipopolysaccharide (LPS) layers. Despite all being Gram-negative, variations in the LPS structure can result in differential susceptibility to antibacterial agents [30].

Biofilms are organized bacterial communities encased in a matrix of self-secreted extracellular polymeric substances (EPS) [31]. The development of a biofilm starts when free-floating (planktonic) bacterial cells temporarily adhere to surfaces, either biotic or abiotic surfaces, and progresses to stable attachment and maturation of the community [32]. In the present study, the flower extracts were found to significantly inhibit both the initial adhesion phase and the development of mature biofilms in pathogenic enteric bacteria, with inhibition rates ranging from 53.18% to 100% for initial adherence and 5.25% to 96.90% for established biofilms. The higher efficacy observed against the early adhesion stage may be due to the increased susceptibility of planktonic cells, which are more accessible and vulnerable to antibacterial agents than cells embedded within mature biofilms [33]. Thus, planktonic cells are primarily responsible for initiating the biofilm matrix, targeting them can effectively disrupt biofilm development at its earliest stage [34]. In contrast, mature biofilms present a substantial challenge due to their complex EPS matrix and extended growth periods, which confer high resistance to antimicrobial agents [35]. The biofilm formation process involves not only cell adhesion via pili but also sophisticated cell-to-cell communication through quorum sensing (QS), which facilitates the coordination and maintenance of biofilm communities [36]. Disrupting QS pathways and adhesion mechanisms has been suggested as an effective strategy to prevent biofilm establishment, thereby offering a promising approach for novel therapeutic development [4]. In addition to interfering with adhesion and QS signaling, plant extracts may also reduce the availability of essential nutrients, both inorganic and organic, thereby impairing bacterial attachment and subsequent growth [37].

The antibacterial and anti-biofilm effects of Thai flower extracts are largely attributed to their phytochemical composition. Medicinal plants generally contain diverse secondary metabolites, many of which serve as natural defense agents against microbial pathogens [36]. According to previous studies, *M. ferrea* flowers were found to harbor a variety of bioactive compounds, including alkaloids, glycosides, tannins, phenolics, coumarins, sterols, xanthones, volatile oils, triterpenoids, resins, and saponins. Specific constituents such as α-copaene, germacrene D, β-amyrin, and β-sitosterol were detected, along with newly identified compounds including mesuanic acid, mesuferrols A and B, mesuaxanthones A and B, mesuaferrins A–C, mesuaferrones A and B, mesuarin, and mesuol [38]. Similarly, the phytochemical profile of *M. siamensis* flowers included predominant compounds such as coumarins and mammeasins A and B [39], as well as xanthones, triterpenes, steroids [40], and flavonoids [41]. Notably, vitexin and isovitexin were identified as functional components common to both *M. ferrea* and *M. siamensis* [7]. For *C. ternatea*, anthocyanins, particularly ternatins, were identified as the major phytochemicals [11], in addition to flavonoids such as kaempferol, quercetin, and myricetin [12]. Other constituents included saponins, tannins, glycosides, triterpenoids, steroids, and alkaloids [42]. LC–MS profiling confirmed the chemical diversity among the three Thai flower extracts. *M. ferrea* extract showed biflavonoids such as mesuaferrone A. *M. siamensis* extract contained catechol and luteolin-6-C-glucoside, while *C. ternatea* extract enriched with trigonelline, kaempferol derivatives, and hydroquinidine. These distinct phytochemical profiles contribute to the differences in antioxidant, anti-bacteria, and antibiofilm activities.

Moreover, the compounds are known to exert anti-biofilm effects through multiple mechanisms including inhibition of substrate availability, disruption of cell walls and membranes, interference with bacterial adhesion molecules, protein binding, and interaction with DNA, which may also contribute to antiviral effects [43]. Certain compounds, such as terpenoids, may promote detachment of planktonic cells from the biofilm by compromising membrane integrity, leading to effective eradication of biofilm-associated cells. Terpenoids are believed to alter the fatty acid composition of bacterial membranes, reducing surface hydrophobicity and thereby interfering with biofilm formation [36]. In comparison, the standard antibiotic gentamicin, used as a positive control, exhibited potent antibacterial and anti-biofilm activities at a lower dose. This superior efficacy can be attributed to its purified form, which contrasts with the complex mixtures of bioactive compounds present in crude plant extracts [36]. However, the diverse mechanisms of action demonstrated by the phytochemicals in the plant extracts underscore their potential as complementary or alternative therapeutic agents in the treatment of biofilm-related infections. DMSO was used as a negative control to dissolve the plant extracts, which were initially prepared at 500 mg/mL in 99.9% DMSO. In the various assays, including agar well diffusion, MIC, MBC, time–kill, and biofilm experiments, the final DMSO concentrations varied according to the dilutions of the extracts. Although DMSO at high concentrations may exhibit inhibitory effects on certain bacteria or biofilm formation [44], the specific DMSO concentrations used in each treatment are provided in Table S4 and Figure S2 to account for these potential effects.

The use of Caco-2 cells provides a physiologically relevant model to assess the impact of flower extracts on bacterial adherence to host tissues. Since adhesion is a key virulence factor, evaluating bacterial attachment to intestinal epithelial cells offers valuable insights into host–pathogen interactions [45]. Bacterial adhesion to intestinal epithelial cells represents an essential initial step in the establishment of infection. In the present work, we examined the potential of flower extracts to disrupt this early adhesion process, which is known to contribute significantly to pathogenesis [46]. Pathogenic enteric bacteria were able to adhere to Caco-2 cells due to the presence of adhesin genes. Both fimbrial and non-fimbrial adhesins are essential mediators of bacterial attachment to intestinal cells, contributing to the development of diarrheal diseases [47].

Treatment with several flower extracts significantly reduced the adhesion of pathogenic enteric bacteria to Caco-2 cells. This anti-adhesion effect can be attributed to the phytochemicals present in the extracts, particularly polyphenols. These compounds have been shown to bind to bacterial adhesins and cell walls, disrupt bacterial membranes, inhibit enzymatic activity, and chelate essential metal ions [48]. Additionally, polyphenolic compounds such as rutin, ferulic acid, and gallic acid can interfere with bacterial adhesion by modulating the expression of adhesin-related genes or by directly binding to surface proteins, thereby preventing bacterial attachment to host cell receptors [49,50]. Moreover, these compounds may alter host cell receptors or associated signaling pathways, further hindering bacterial recognition and attachment [51,52]. Further investigations are needed to elucidate the specific mechanisms underlying these effects.

The increasing resistance of bacteria, including *E. coli* O157:H7, to multiple antibiotics poses a significant challenge for treatment and demonstrates the need for alternative antimicrobial strategies [53]. Flower extracts were effective in disrupting biofilms and reducing the adhesion of various pathogenic enteric bacteria at their MIC values. These effects were further confirmed by SEM analysis of *E. coli* O157:H7, which revealed significant damage to the bacterial cell walls. In the presence of flower extracts, *E. coli* O157:H7 cells exhibited notable morphological alterations, including irregular, shriveled, and rugged surfaces, indicating a loss of structural integrity. These observations suggest that flower extracts can induce substantial disruption of the cell wall, ultimately leading to bacterial cell damage. Similarly, Zulkamal et al. [54] reported that *C. ternatea* aqueous extract induced morphological changes in *Streptococcus mutans*, resulting in the loss of the original bacterial shape and severe structural damage, including crumpling, cavitation, and irregular forms, ultimately leading to cell death. *M. ferrea* leaf and fruit extracts caused direct alterations in the morphology of *S. aureus*, affecting membrane permeability and integrity. Additionally, exposure to high levels of gallic acid induced irregularities in cell morphology in *E. coli* 3110 [55]. Although the exact mechanisms remain unclear, it has been suggested that the active components of the extracts may disrupt the bacterial cytoplasmic membrane by binding to the cell surface and penetrating the phospholipid bilayer, leading to damage to both the cell wall and membrane [56].

To further investigate the effects of flower extracts on bacterial membrane permeability, the levels of extracellular DNA, RNA, and proteins were measured in *E. coli* O157:H7. An increase in the leakage of these intracellular components indicated compromised membrane integrity following exposure to the extracts [57]. Damage to bacterial membranes leads to the release of essential ions such as K^+^ and (PO_4_)^3−^, along with larger molecules like DNA and RNA [58]. The leakage of nucleic acids and proteins serves as a critical marker of membrane disruption. Previous studies have demonstrated that flavonoids and gallic acid can induce membrane perforation, inhibit nucleic acid synthesis, interfere with energy metabolism, and ultimately compromise the cell integrity of *E. coli* and *S.* Typhimurium [59,60]. Flavonoids interact with DNA primarily through non-covalent bonds, and the overall charge of the flavonoid–DNA complex can be affected by the type of interaction and the surrounding pH. Depending on these factors, the complex may carry a positive or negative charge [61]. This interaction can result in drastic impacts on bacterial DNA, potentially inhibiting bacterial viability [62]. Moreover, hyperoside and quercetin disrupted the cell membrane integrity of *Pseudomonas aeruginosa* and *Shewanella putrefaciens*, causing ion leakage and increased medium conductivity. SEM analysis revealed morphological damage, including shrinkage and pore formation. Both compounds inhibited motility, EPS production, and biofilm formation. They also interacted with or degraded bacterial DNA, with molecular docking suggesting binding to AT-rich minor grooves, contributing to bacterial death [63]. Therefore, it is plausible that the antibacterial activity of the flower extracts against *E. coli* O157:H7 may be attributed to flavonoids, including hyperoside and quercetin, which promote membrane damage and cellular leakage. However, several limitations must be considered in this study. First, the precise molecular mechanisms underlying the antibacterial and anti-biofilm activities of the flower extracts remain unclear. Future studies should focus on identifying and characterizing specific active compounds and their interactions with bacterial targets. Additionally, while this study used in vitro models, further research using in vivo models would provide more physiologically relevant data on the effectiveness of these extracts in real-world applications. Finally, the stability and bioavailability of the flower extracts need to be evaluated to assess their potential for use in clinical or pharmaceutical settings. Further investigations into these areas will provide a more comprehensive understanding of the therapeutic potential of these natural compounds.

## 4. Materials and Methods

### 4.1. Plant Material

The dried flowers of *M. ferrea* L. (Bunnak), *M. siamensis* T. Anderson (Saraphi), and *C. ternatea* (Anchan) were purchased from the Lanna Herbal Shop in Chiang Mai Province, northern Thailand. The plant materials were taxonomically identified by Dr. Wittaya Pongamornkul, an ethnobotanist and botanist at the Research and Conservation Department, Queen Sirikit Botanic Garden Herbarium (QSBG Herbarium). Voucher specimens of *M. ferrea* L. (WP9500), *M. siamensis* T. Anderson (WP9501), and *C. ternatea* (WP9502) were deposited at the Queen Sirikit Botanic Garden Herbarium (QSBG), Chiang Mai, Thailand.

### 4.2. Preparation of Flower Samples and Extraction Procedure

**Figure 12:**
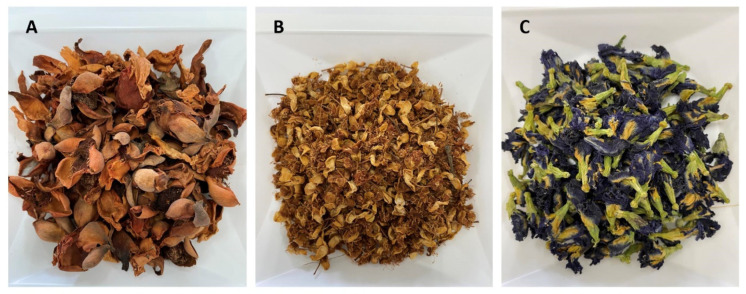
Dried flowers of (**A**) *Mesua ferrea* L. (Bunnak), (**B**) *Mammea siamensis* T. Anderson (Saraphi), and (**C**) *Clitoria ternatea* (Anchan).

The extraction procedure was performed using the maceration technique [64]. The dried flowers of *M. ferrea* L., *M. siamensis*, and *C. ternatea* (Figure 12) were ground into a fine powder. For the extraction, 200 g of flower powder was mixed with 2 L of 95% ethanol (1:10 *w*/*v*) in a 2.5 L container. The mixture was stirred on an orbital shaker (IKA, Staufen, Germany) at 150 rpm at room temperature for 72 h. After extraction, the solution was filtered through Whatman No. 1 filter paper. The filtrate was then concentrated under reduced pressure at 45 °C using a rotary evaporator (Heidolph, Schwabach, Germany) and freeze-dried (LABCONCO, Kansas City, MO, USA). The resulting crude extracts were weighed to determine yield and dissolved in 99.9% DMSO (RCI Labscan, Bangkok, Thailand) to a final concentration of 500 mg/mL for subsequent experiments.

### 4.3. Identification of Bioactive Compounds in Flower Extracts by HPLC Analysis

The levels of gallic acid and quercetin in the flower extracts were analyzed using high-performance liquid chromatography (HPLC) following slight modifications of published protocols [65,66]. An Agilent 1260 Infinity II system (Santa Clara, CA, USA) with a C-18 column (4.6 × 150 mm, 5 µm; GL Sciences, Tokyo, Japan) maintained at 40 °C was used. The mobile phase consisted of 1% formic acid in water (solvent A) and acetonitrile (solvent B). A gradient program was applied: from 0 to 30 min, solvent A decreased linearly from 90% to 70%, and from 30 to 40 min, it increased from 70% to 95%. The flow rate was 1.0 mL/min, with detection at 254 nm.

**Figure 13:**
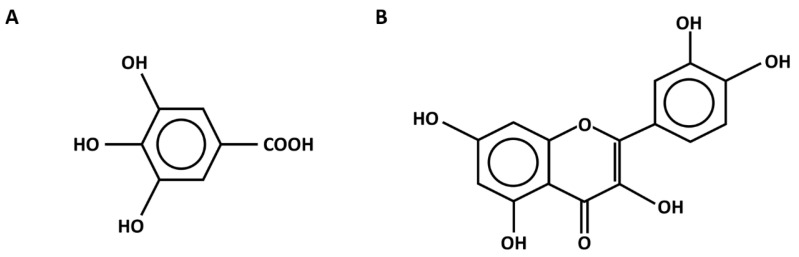
Chemical structure of (**A**) gallic acid and (**B**) quercetin.

Prior to injection, the flower extracts were dissolved in a methanol–water mixture (6:4, *v*/*v*) and filtered through a 0.45 µm sterile syringe filter. Standard solutions of gallic acid (Sigma-Aldrich, St. Louis, MO, USA) and quercetin (Merck, Billerica, MA, USA) were also prepared under the same conditions. Compounds in the extracts were identified by comparing their retention times with those of the standards (Figure 13), and concentrations were calculated using standard calibration curves.

### 4.4. Identification of Bioactive Compounds in Flower Extracts by LC-MS Analysis

The chemical constituents of the flower extracts were characterized using liquid chromatography coupled with quadrupole time-of-flight mass spectrometry (LC-QTOF-MS; 6545XT AdvanceBio LC/Q-TOF, Agilent Technologies, Santa Clara, CA, USA) [67]. Chromatographic separation was performed on an InfinityLab Poroshell 120 EC-C18 column (2.1 × 100 mm, 2.7 µm, 120 Å; Agilent Technologies, Santa Clara, CA, USA) maintained at 50 °C. The mobile phase consisted of 0.1% (*v*/*v*) formic acid in water (solvent A) and 0.1% (*v*/*v*) formic acid in acetonitrile (solvent B). A gradient elution was applied over 20 min at a flow rate of 0.4 mL/min, with an injection volume of 10 µL.

Mass spectrometry detection was conducted under both positive and negative electrospray ionization (ESI) modes using high-resolution settings. The ion source parameters were as follows: drying gas at 325 °C and 13 L/min, sheath gas at 275 °C and 12 L/min, nebulizer pressure at 45 psi, and capillary voltage of +4000 V/−3000 V. The instrument was set to scan over an *m*/*z* range of 40–1700 (MS1) and 25–1000 (MS2), with collision energies of 20 eV in positive and 10 eV in negative modes.

Data acquisition and processing were performed using MS-DIAL version 5.3 (RIKEN CSRS, Yokohama, Kanagawa, Japan). Sulfadimethoxine served as the internal standard for normalization, and metabolite identification was based on comparison with the in-house ESI(+/−) MS/MS spectral library of authentic standards provided in MS-DIAL.

### 4.5. Determination of Total Phenolic and Flavonoid Contents in Flower Extracts

The Folin–Ciocalteu assay [68] was employed to determine total phenolic content in the flower extracts. In brief, 20 µL of each extract was added to 10 µL of 50% Folin–Ciocalteu reagent, followed by 100 µL of distilled water and 20 µL of 95% ethanol. After allowing the reaction to proceed for 5 min, 20 µL of 5% (*w*/*v*) sodium carbonate was incorporated. The mixture was incubated for 1 h at room temperature, after which the absorbance was measured at 725 nm using a microplate spectrophotometer (DYNEX SPECTRA MR, Chantilly, VA, USA). Phenolic content was quantified against a gallic acid standard curve (10–100 µg/mL) and expressed as milligrams of gallic acid equivalents per gram of extract (mg GAE/g extract).

The aluminum chloride colorimetric assay [69] was employed to quantify total flavonoids in the flower extracts. Briefly, 20 µL of each extract was combined with 4 µL of 10% aluminum chloride, 60 µL of methanol, 4 µL of 1 M potassium acetate, and 112 µL of distilled water. The reaction mixture was allowed to incubate for 30 min at room temperature, and absorbance was measured at 415 nm using a microplate spectrophotometer. Total flavonoid content was calculated using a quercetin standard curve (7.81–125 µg/mL) and expressed as mg quercetin equivalents per gram of extract (mg QE/g extract).

### 4.6. Antioxidant Activities

#### 4.6.1. DPPH Radical Scavenging Assay

The DPPH assay [70] was used to evaluate the radical scavenging activity of flower extracts with minor modifications. In brief, a 0.1 mM DPPH solution in methanol (Sigma-Aldrich, St. Louis, MO, USA) was prepared, and 150 µL of this solution was added to 50 µL of each extract. The mixture was gently mixed and incubated in the dark for 20 min at room temperature. Absorbance was read at 517 nm using a microplate spectrophotometer. The antioxidant capacity was expressed as IC_50_, representing the concentration of extract required to neutralize 50% of DPPH radicals, and was calculated against a gallic acid standard curve (1–8 µg/mL), with results reported as mg GAE/g extract.

#### 4.6.2. ABTS Radical Cation Decolorization Assay

The ABTS•^+^ assay [71] was used to determine the radical cation decolorization activity of flower extracts with slight modifications. Briefly, ABTS•^+^ stock solution was prepared by reacting 7 mM ABTS diammonium salt with 2.45 mM potassium persulfate and incubating in the dark for 12–16 h. The solution was then diluted with distilled water to an absorbance of 0.700 ± 0.020 at 734 nm. A total of 195 µL of ABTS•^+^ solution was added to 5 µL of each extract, mixed gently, and incubated in the dark for 10 min at room temperature. Absorbance was measured at 734 nm using a microplate spectrophotometer. The antioxidant capacity was expressed as IC_50_, representing the extract concentration required to quench 50% of ABTS•^+^ radicals, and calculated based on a Trolox standard curve (25–300 µg/mL), with results reported as milligrams of Trolox equivalent antioxidant capacity per gram of extract (mg TEAC/g extract).

#### 4.6.3. Ferric Reducing Antioxidant Power (FRAP) Assay

The FRAP assay [72] was used to evaluate the ferric reducing antioxidant power of flower extracts. The FRAP working reagent was prepared by mixing 10 mL of 300 mM acetate buffer (pH 3.6), 1 mL of 10 mM TPTZ (2,4,6-tri(2-pyridyl)-s-triazine) (Merck, Billerica, MA, USA) in 40 mM HCl, and 1 mL of 20 mM ferric chloride solution. A total of 150 µL of FRAP reagent was added to 5 µL of each extract, mixed gently, and incubated in the dark for 15 min at room temperature. Absorbance was measured at 593 nm using a microplate spectrophotometer. Antioxidant capacity was calculated against a ferrous sulfate (FeSO_4_) standard curve (10–100 µg/mL) and reported as mg milligrams of ferrous sulfate (FeSO_4_) equivalent per gram of extract (mg FeSO_4_/g extract).

### 4.7. Antibacterial Activities

#### 4.7.1. Bacterial Strains

Pathogenic enteric bacteria used in this study; *E. coli* and *V. cholerae* were obtained from the Division of Microbiology, Department of Medical Technology, Faculty of Associated Medical Sciences, whereas *E. coli* O157:H7 DMST 12743, *S.* Typhi DMST 22842, and *S. dysenteriae* DMST 1511 were obtained from the SCB 2711 Microbiology Laboratory. These strains were utilized to evaluate the antibacterial activity of flower extracts.

#### 4.7.2. Agar Well Diffusion Method

The antibacterial activity of flower extracts was evaluated using the agar well diffusion method [26]. Bacterial strains, including *E. coli*, *E. coli* O157:H7, *S.* Typhi, *S. dysenteriae*, and *V. cholerae*, were adjusted to a turbidity equivalent to a 0.5 McFarland standard (CLSI M07-A10) [28] and then swabbed onto Mueller–Hinton agar (MHA) plates (HiMedia, Mumbai, Maharashtra, India). A hole with an 8 mm diameter was aseptically punched using a sterile cork borer, and 100 µL of the flower extract was transferred into each well. Gentamicin (1 mg/mL; Bio Basic Inc., Markham, ON, Canada) was used as the positive control, and 99.9% DMSO served as the negative control. Plates were incubated at 37 °C for 24 h, and antimicrobial activity was evaluated by measuring the inhibition zone diameters (mm) for each bacterial species. All assays were performed in triplicate.

#### 4.7.3. Minimum Inhibitory Concentration (MIC) and Minimum Bactericidal Concentration (MBC)

The inhibitory effect of flower extracts on bacterial growth was determined using the broth microdilution method, as indicated by the Clinical and Laboratory Standards Institute of the United States of America (CLSI M07-A10) [28]. Flower extracts were diluted in Mueller–Hinton broth (MHB) by two-fold serial dilution and added to each well of a sterile, round-bottom 96-well culture plate (100 μL/well; SPL Life Sciences, Pocheon-si, Gyeonggi-do, Korea). The bacterial suspension, previously adjusted to a 0.5 McFarland standard and containing approximately 1–2 × 10^8^ colony-forming units (CFU)/mL, was appropriately diluted to obtain a final concentration of 10^6^ CFU/mL. Then, 100 µL of this suspension was inoculated into each well, yielding a final bacterial concentration of approximately 10^5^ CFU/mL. The plates were incubated at 37 °C for 24 h. After incubation, the minimum inhibitory concentration (MIC) was determined as the lowest extract concentration that visibly inhibited bacterial growth [73].

To determine the minimum bactericidal concentration (MBC), viable cells from wells showing no visible bacterial growth were streaked onto agar plates, followed by incubation at 37 °C for 24 h. The MBC endpoint was defined as the lowest concentration of the flower extract that resulted in a 99.9% reduction in the bacterial population [73].

#### 4.7.4. Time–Kill Assay

The bactericidal effect of flower extracts was evaluated using a time–kill assay [74]. Bacterial suspension was adjusted to a 0.5 McFarland standard and diluted to approximately 10^5^ CFU/mL. The bacterial suspension was treated with flower extracts at concentrations equal to the MIC and incubated at 37 °C. Samples were collected at 0, 2, 4, 6, 12, and 24 h, serially diluted in phosphate-buffered saline (PBS), and 100 µL of each dilution was spread onto Mueller–Hinton agar plates using a glass bead method [75]. Following incubation at 37 °C for 24 h, colonies were counted, and bacterial viability was expressed as log_10_ CFU/mL relative to the initial inoculum. All experiments were performed in triplicate.

### 4.8. Anti-Biofilm Assay

#### 4.8.1. Effects on Adherence Biofilm

The effects of flower extracts on adherent biofilms were assessed using the crystal violet staining method [76]. A 100 μL bacterial inoculum was adjusted to an initial optical density at 600 nm (OD_600_) of 0.01 in BD Bacto™ tryptic soy broth (TSB; Becton, Dickinson and Company, Franklin Lakes, NJ, USA) supplemented with 1% glucose and added to 96-well plates. Then, 100 μL of the flower extract was distributed into the wells at a final concentration of 1 MIC. The plates were incubated at 37 °C for 24 h. After incubation, the medium was discarded, and the wells were washed with PBS. Adherent cells were then fixed and stained with 0.1% crystal violet (PanReac AppliChem, Barcelona, Spain) for 10 min. The wells were washed twice with sterile distilled water to remove excess stains. The plates were air-dried to fix the biofilms, which were subsequently quantified by resolubilizing the crystal violet in 200 μL of 33% acetic acid for 5 min. Absorbance was measured at 595 nm using a microplate spectrophotometer [77]. The inhibitory effect of each extract was expressed as a percentage relative to the untreated control using the following formula:

$$Relative\;inhibition\;\left(\%\right)=\frac{Absorbance\;control-Absorbance\;treatment}{Absorbance\;control}\times 100$$

#### 4.8.2. Effects on Established Biofilm

The effects of flower extracts on established biofilms were assessed using the crystal violet staining method [76]. The bacterial inoculum was adjusted to an OD_600_ of 0.01 in BD Bacto™ TBS supplemented with 1% glucose. Then, 200 μL aliquots of the inoculum were transferred to 96-well plates and incubated at 37 °C to allow biofilm formation for 24 h. After removing the planktonic cells, 200 μL of the flower extracts at a concentration of 1 MIC were added to the wells and incubated at 37 °C for 24 h [78]. The biofilms were then fixed and stained with 0.1% crystal violet for 10 min. The wells were washed twice with sterile distilled water to remove excess stains. The plates were air-dried to fix the biofilms, which were subsequently quantified by resolubilizing the crystal violet in 200 μL of 33% acetic acid for 5 min. Biofilm biomass was then measured at 595 nm using a microplate spectrophotometer, following the same procedure as described above in Section 4.8.1.

### 4.9. Antibacterial Adhesion Activity on Caco-2 Cells

#### 4.9.1. Cell Culture and Cytotoxicity by Flower Extracts

Caco-2 human colorectal carcinoma cells were cultured in Dulbecco’s Modified Eagle’s Medium (DMEM; Gibco, Grand Island, NY, USA) supplemented with 10% heat-inactivated fetal bovine serum (FBS) and 1% penicillin–streptomycin (100 U/mL penicillin and 100 µg/mL streptomycin; Gibco, Grand Island, NY, USA) at 37 °C in a 5% CO_2_ incubator [79]. Cytotoxicity of the flower extracts was assessed using the MTT assay according to standard protocols [80].

#### 4.9.2. Determination of Antibacterial Adhesion by Flower Extracts

The efficacy of the flower extracts in inhibiting bacterial adhesion to Caco-2 cells was determined using the antibacterial adhesion assay [77]. A cell suspension at 1 × 10^5^ cells/mL was transferred to 24-well cell culture plates and incubated in a 5% CO_2_ atmosphere at 37 °C for 24 h. Afterward, the medium was discarded, and a bacterial suspension grown to mid-log phase in diluted medium was added to the 24-well plates at a multiplicity of infection (MOI) of 100. Then, flower extracts at a non-toxic concentration were immediately added to the co-culture. After co-culture for 1 h, unbound bacteria were removed by washing with PBS. Adherent bacteria were detached with 0.1% Triton X-100 and quantified by viable plate counting [75]. The relative percentage of antibacterial adhesion was calculated as

$$Relative\;antibacterial\;adhesion\;\left(\%\right)=\frac{N\;control-N\;treatment}{N\;control}\times 100$$

where N control is the number of bacteria attached in the untreated control, and N treatment is the number attached in wells treated with flower extracts.

### 4.10. Cellular Structure of E. coli O157:H7

#### 4.10.1. SEM Analysis

Morphological changes in *E. coli* O157:H7 after treatment with flower extracts were observed by scanning electron microscopy (SEM) [81,82]. The bacterial inoculum was adjusted to an OD_600_ of 0.01 and added to 24-well plates containing glass coverslips coated with 0.2% gelatin [83]. Flower extracts at a concentration of 1 and 1/2 MIC were added to the wells, and the plates were incubated at 37 °C for 24 h. The bacterial specimens were fixed with 2.5% (*v*/*v*) glutaraldehyde, followed by incubation with 1.0% osmium tetroxide (OsO_4_). The bacterial samples were progressively dehydrated using increasing concentrations of ethanol, followed by drying at the critical point with CO_2_. The dried specimens were attached to aluminum stubs, covered with a thin layer of gold, and visualized using a Hitachi SU3800 scanning electron microscope (Minato-ku, Tokyo, Japan).

#### 4.10.2. Cell Membrane Damage Assays

Assays for nucleic acid (DNA and RNA) and protein leakage were conducted to evaluate the destructive effect of the flower extract on the *E. coli* O157:H7 cell membrane [57,84]. A 10 mL suspension of 24 h *E. coli* O157:H7 culture was centrifuged at 3500 rpm for 15 min. The resulting pellets were washed three times with PBS, after which 10 mL of PBS was added and mixed with the flower extracts at concentrations of 1 and 1/2 MIC. The mixture was incubated at 37 °C for 24 h, followed by centrifugation at 3500 rpm for 15 min. The supernatant was then used to assess cell membrane leakage. DNA and RNA released from the cytoplasm were quantified at 260 nm using a NanoDrop One^c^ Microvolume UV-Vis Spectrophotometers (Thermo Scientific, Waltham, MA, USA). Protein levels in the collected supernatants were determined using a BCA protein assay kit (EMD Millipore, Burlington, MA, USA). Absorbance was recorded at 562 nm, and bovine serum albumin (BSA) was employed as the calibration standard.

### 4.11. Statistical Analysis

All experiments were conducted in triplicate, and the results are presented as mean ± standard deviation (SD). Statistical comparisons among groups were performed using one-way analysis of variance (ANOVA) followed by Tukey’s honestly significant difference (HSD) post hoc test. Differences with *p* < 0.05 were considered statistically significant. Analyses were carried out using SPSS software (version 17.0, IBM Corp., Chicago, IL, USA). For the analysis of DNA, RNA, and protein contents, comparisons were performed exclusively between flower extract-treated samples and the untreated *E. coli* O157:H7 control group, without intergroup comparisons among treated samples.

## 5. Conclusions

This study demonstrated that Thai flower extracts are rich sources of phenolic and flavonoid compounds, which significantly contribute to their antioxidant activities, as evidenced by the DPPH, ABTS, and FRAP assays. LC–MS profiling further revealed the presence of specific bioactive metabolites. Despite only detecting gallic acid and quercetin through HPLC analysis, the crude extracts exhibited marked antibacterial effects against pathogenic enteric bacteria, including inhibition of biofilm formation, eradication of established biofilms, and reduction in bacterial adhesion to intestinal epithelial cells. The antibacterial mechanism, particularly against *E. coli* O157:H7, appears to involve damage to the bacterial cell wall and membrane integrity, as well as interference with nucleic acid and protein synthesis. These findings highlight the promising potential of these flower extracts as natural antibacterial agents. However, further studies, including detailed mechanistic investigations, in vivo evaluations, and assessments of stability and bioavailability, are necessary to fully elucidate their therapeutic potential, safety profiles, and practical applicability in clinical settings.

## Figures and Tables

**Table 1:** Percentage yields of flower extracts.

Flower Extracts	Yield (%)
*Mesua ferrea*	18.91
*Mammea siamensis*	16.03
*Clitoria ternatea*	17.01

**Table 2:** Phytochemicals compounds of flower extracts by HPLC analysis.

Flower Extracts	Gallic Acid (mg/g Extract)	Quercetin (mg/g Extract)
*Mesua ferrea*	16.956 ± 0.059 ^b^	0.260 ± 0.027 ^a^
*Mammea siamensis*	0.921 ± 0.015 ^a^	0.678 ± 0.025 ^b^
*Clitoria ternatea*	ND	ND

ND; non-detected. Statistical significance was denoted by different letters (^a,b^) indicating a significant difference *(p* < 0.05).

**Table 3:** Total phenolic and flavonoid content in flower extracts.

Flower Extracts	Total Phenolic Content (mg GAE/g Extract)	Total Flavonoid Content (mg QE/g Extract)
*Mesua ferrea*	50.09 ± 1.01 ^c^	12.48 ± 0.48 ^a^
*Mammea siamensis*	26.02 ± 0.62 ^b^	16.51 ± 0.01 ^b^
*Clitoria ternatea*	10.66 ± 0.85 ^a^	19.37 ± 0.91 ^c^

Statistical significance was denoted by different letters (^a–c^) indicating a significant difference (*p* < 0.05).

**Table 4:** Evaluation of antioxidant activities of flower extracts using DPPH, ABTS, and FRAP assays.

Flower Extracts	DPPH		ABTS		FRAP
	IC_50_ (mg/mL)	Antioxidant Activity (mg GAE/g Extract)	IC_50_ (mg/mL)	Antioxidant Activity (mg TEAC/g Extract)	Antioxidant Activity (mg FeSO_4_/g Extract)
*Mesua ferrea*	2.09 ± 0.06 ^c^	2.31 ± 0.14 ^a^	10.96 ± 1.04 ^c^	15.78 ± 1.59 ^a^	186.49 ± 9.36 ^c^
*Mammea siamensis*	0.47 ± 0.02 ^b^	10.20 ± 0.25 ^b^	2.87 ± 0.28 ^b^	60.29 ± 5.81 ^b^	81.03 ± 4.02 ^b^
*Clitoria ternatea*	0.30 ± 0.03 ^a^	16.48 ± 2.00 ^c^	1.00 ± 0.14 ^a^	159.46 ± 3.21 ^c^	37.45 ± 3.11 ^a^

Statistical significance was denoted by different letters (^a–c^) indicating a significant difference (*p* < 0.05).

**Table 5:** Antibacterial activity of flower extracts against some pathogenic enteric bacteria.

Flower Extracts (500 mg/mL)	Inhibition Zone Diameter (mm)				
	*E. coli*	*E. coli* O157:H7	*S.* Typhi	*S. dysenteriae*	*V. cholerae*
*Mesua ferrea*	13.00 ± 0.87 ^cd^	13.33 ± 1.15 ^cd^	15.00 ± 0.00 ^d^	14.00 ± 2.65 ^d^	14.83 ± 1.04 ^d^
*Mammea siamensis*	10.00 ± 0.00 ^b^	0.00 ^a^	11.00 ± 1.73 ^bc^	10.67 ± 1.53 ^bc^	0.00 ^a^
*Clitoria ternatea*	0.00 ^a^	0.00 ^a^	0.00 ^a^	0.00 ^a^	0.00 ^a^
Gentamicin (1 mg/mL)	26.67 ± 0.58 ^e^	29.33 ± 0.58 ^f^	26.67 ± 0.58 ^e^	28.33 ± 0.58 ^ef^	26.67 ± 0.58 ^e^

Data in the table are presented as mean ± standard deviation (*n* = 3) for flower extracts at 500 mg/mL. Inhibition zone diameters (mm) were recorded using the agar well diffusion assay. Statistical significance was evaluated at *p* < 0.05, with distinct letters (^a–f^) denoting significant differences among groups. Gentamicin (1 mg/mL) served as the positive control.

**Table 6:** Evaluation of antibacterial effects of flower extracts based on MIC and MBC.

Flower Extracts	Concentration of Flower Extracts (mg/mL)									
	*E. coli*		*E. coli* O157:H7		*S.* Typhi		*S. dysenteriae*		*V. cholerae*	
	MIC	MBC	MIC	MBC	MIC	MBC	MIC	MBC	MIC	MBC
*Mesua ferrea*	62.5	62.5	62.5	62.5	31.25	31.25	31.25	31.25	31.25	31.25
*Mammea siamensis*	62.5	62.5	62.5	62.5	62.5	62.5	31.25	31.25	62.5	62.5
*Clitoria ternatea*	125	125	125	125	125	125	125	125	125	125
Gentamicin	0.0078	0.0078	0.0078	0.0078	0.0078	0.0078	0.0078	0.0078	0.0078	0.0078

**Table 7:** Inhibition of bacterial adhesion to intestinal epithelial cells by flower extracts.

Flower Extracts	Inhibition of Bacterial Adhesion (%)				
	*E. coli*	*E. coli* O157:H7	*S.* Typhi	*S. dysenteriae*	*V. cholerae*
*Mesua ferrea*	20.49 ± 1.78 ^de^	24.08 ± 0.36 ^e^	3.14 ± 1.22 ^a^	5.13 ± 0.52 ^ab^	12.43 ± 1.41 ^bc^
*Mammea siamensis*	16.06 ± 0.95 ^cd^	24.78 ± 3.61 ^e^	57.41 ± 0.21 ^g^	6.41 ± 0.26 ^ab^	24.29 ± 6.06 ^e^
*Clitoria ternatea*	16.11 ± 3.53 ^cd^	38.61 ± 0.39 ^f^	55.18 ± 0.54 ^g^	4.76 ± 2.07 ^ab^	17.86 ± 1.01 ^cde^

Statistical significance was denoted by different letters (^a–g^) indicating a significant difference (*p* < 0.05).

## Data Availability

The original contributions presented in this study are included in the article/Supplementary Materials; further inquiries can be directed to the corresponding author.

## Supplementary Materials

The following supporting information can be downloaded at https://www.mdpi.com/article/10.3390/antibiotics14101038/s1. Figure S1: Effect of flower extracts on the viability of Caco-2 cells; Figure S2: Effect of DMSO on time kill assay of flower extracts; Table S1: Putative metabolites detected in flower extracts by LC–MS, showing retention time, mass-to-charge ratio, adduct ion, identification score, and relative abundance; Table S2: Inhibition of bacterial growth (%) as determined by time–kill assay; Table S3: Inhibition of adherence biofilm and established biofilm; Table S4: Inhibition of established biofilm; Table S5: Effect of DMSO on antibacterial activity of flower extracts; Table S6. IC50 values and selectivity index (SI) of flower extracts.

## Abbreviations

The following abbreviations are used in this manuscript:

ABTS	2,20-azinobis-(3-ethylbenzothiazolin-6-sulfonic acid
DPPH	2,2-diphenyl-1-picrylhydrazil
FRAP	ferric reducing antioxidant power
GAE	gallic acid equivalent
HPLC	high-performance liquid chromatography
LC-MS	liquid chromatography–mass spectrometry
MIC	minimum inhibitory concentration
MBC	minimum bactericidal concentration
QE	quercetin equivalent
TEAC	trolox equivalent antioxidant capacity
