# Supplementary material for: Thai Medicinal Flowers as Natural Antioxidants and Antibacterial Agents Against Pathogenic Enteric Bacteria: A Comparative Study of Mesua ferrea, Mammea siamensis, and Clitoria ternatea
Source: Antibiotics (Basel). 2025 Oct 16;14(10):1038. doi: 10.3390/antibiotics14101038 (PMC12561062; doi:10.3390/antibiotics14101038)
Supplement: Supplementary file 1 [file antibiotics-14-01038-s001.zip › antibiotics-3924716-supplementary.pdf]

Supplementary Materials

Table S1. Putative metabolites detected in flower extracts by LC–MS, showing retention time, mass-to-charge ratio, adduct ion, identification score, and relative abundance.

**Putative metabolite name**	**Molecular formula**	**RT (min)**	**m/z**	**Adduct ion**	**Score**	**Relative abundance of metabolite**		
						M. ferrea	***M. siamensis***	***C. ternatea***
3,4-Dihydroxybenzoic acid	$C_7H_6O_4$	2.752	153.01971	$[M-H]^-$	1.63	10696444.24	6992510.43	27322.98
Catechin	$C_{15}H_{14}O_6$	4.125	289.07242	$[M-H]^-$	1.57	4987210.77	499039.88	2131.64
Catechol	$C_6H_6O_2$	2.710	109.03387	$[M-H]^-$	1.66	28651754.69	25215828.96	118592.23
Hydroquinidine	$C_{20}H_{26}N_2O_2$	12.466	325.19519	$[M-H]^-$	1.53	0.00	0.00	15,009,556.38
Isoorientin	$C_{21}H_{20}O_{11}$	0.742	449.1069	$[M+H]^+$	1.5	600908.78	492150.15	115302.87
Kaempferol	$C_{15}H_{10}O_6$	5.039	287.05533	$[M+H]^+$	1.73	33167.06	26679.69	5114676.11
Kaempferol-3-glucoside	$C_{21}H_{20}O_{11}$	5.072	449.10706	$[M+H]^+$	1.53	122677.22	10224.07	793392.37
Luteolin-6-C-glucoside	$C_{21}H_{20}O_{11}$	4.631	447.09454	$[M-H]^-$	1.56	11307118.00	21630942.13	50506.39
Mesuaferrone A	$C_{30}H_{22}O_{10}$	8.266	543.12701	$[M+H]^+$	1.6	6189111.39	291607.81	2974.05
Nootkatone	$C_{15}H_{22}O$	12.583	219.17387	$[M+H]^+$	1.64	3875510.86	12398.25	7730.28

Quercetin	$C_{15}H_{10}O_7$	6.716	301.03534	$[M-H]^-$	1.55	1,899,591.48	8,286,978.18	427,310.53
Quercitrin	$C_{21}H_{20}O_{11}$	5.507	447.09677	$[M-H]^-$	1.62	10406809.29	3167540.06	1413409.85
Rutin	$C_{27}H_{30}O_{16}$	4.927	609.14740	$[M-H]^-$	1.56	422666.09	8760.87	3289935.71
Trigonelline	$C_7H_7NO_2$	0.743	138.05476	$[M+H]^+$	1.64	45894.97	778630.36	5866729.33
Gallic acid	$C_7H_6O_5$	0.743	169.01425	$[M-H]^-$	1.49	6,818,137.60	824,083.76	10,218.31

Table S2. Inhibition of bacterial growth (%) as determined by time-kill assay.

A. *Escherichia coli*

Hours	Inhibition of bacterial growth (%)			
	Gentamicin	*Mammea siamensis*	*Clitoria ternatea*	*Mesua ferrea*
**0**	12.19	0.00	0.00	0.00
**2**	100.00	24.58	23.95	21.74
**4**	100.00	37.33	36.52	36.17
**6**	100.00	42.88	43.66	40.80
**12**	100.00	43.58	44.50	44.07
**24**	100.00	79.54	68.00	67.91

B. *Escherichia coli* O157:H7

Hours	Inhibition of bacterial growth (%)			
	Gentamicin	*Mammea siamensis*	*Clitoria ternatea*	*Mesua ferrea*
**0**	19.67	0.00	0.06	0.68
**2**	100.00	18.04	16.58	17.65
**4**	100.00	30.12	29.22	28.84
**6**	100.00	35.88	38.10	30.73
**12**	100.00	48.03	47.69	48.30
**24**	100.00	100.00	74.56	100.00

C. *Salmonella* Typhi

Hours	Inhibition of bacterial growth (%)			
	Gentamicin	*Mammea siamensis*	*Clitoria ternatea*	*Mesua ferrea*
**0**	20.95	0.00	0.00	0.00
**2**	100.00	19.59	17.07	15.36
**4**	100.00	37.61	33.34	27.28
**6**	100.00	64.17	53.15	39.01
**12**	100.00	100.00	69.02	35.21
**24**	100.00	100.00	100.00	37.20

D. ***Shigella dysenteriae***

Hours	Inhibition of bacterial growth (%)			
	**Gentamicin**	***Mammea siamensis***	***Clitoria ternatea***	***Mesua ferrea***
**0**	24.03	0.00	3.90	0.00
**2**	100.00	13.47	21.86	14.12
**4**	100.00	27.52	33.29	27.01
**6**	100.00	45.90	50.34	46.61
**12**	100.00	46.89	68.64	46.42
**24**	100.00	55.55	100.00	47.80

E. ***Vibrio cholerae***

Hours	Inhibition of bacterial growth (%)			
	**Gentamicin**	***Mammea siamensis***	***Clitoria ternatea***	***Mesua ferrea***
**0**	18.09	0.27	0.00	0.43
**2**	100.00	19.44	17.25	16.51
**4**	100.00	36.03	36.05	26.72
**6**	100.00	42.02	43.51	30.52
**12**	100.00	50.49	43.20	26.11
**24**	100.00	100.00	70.49	36.93

Table S3. Inhibition of adherence biofilm.

Flower extracts	Inhibition of adherence biofilm (%)				
	E. coli	*E. coli* O157:H7	*S.* Typhi	*S. dysenteriae*	*V. cholerae*
Mesua ferrea	92.50±1.97	53.18±3.83	96.20±3.38	97.00±1.88	81.78±2.69
Mammea siamensis	97.60±0.63	100.00±2.95	93.39±2.24	98.45±2.67	94.41±1.54
Clitoria ternatea	98.15±2.64	84.11±5.99	94.80±2.65	100.00±5.05	93.38±2.85
**Gentamicin**	97.60±0.10	94.71±0.63	97.21±2.97	96.97±2.46	98.43±1.45

Table S4. Inhibition of established biofilm.

Flower extracts	Inhibition of established biofilm (%)				
	E. coli	*E. coli* O157:H7	*S.* Typhi	*S. dysenteriae*	*V. cholerae*
Mesua ferrea	68.47±0.87	96.90±1.80	56.97±1.44	23.49±3.51	92.68±4.61
Mammea siamensis	20.76±2.10	95.90±0.04	78.19±2.07	19.61±2.08	100.00±0.64
Clitoria ternatea	82.90±0.48	90.54±3.83	93.14±2.01	5.25±0.83	69.95±2.04
**Gentamicin**	22.82±0.51	42.98±1.89	23.54±1.71	18.21±2.78	88.81±0.07

Table S5. Effect of DMSO on antibacterial activity of flower extracts.

A. Agar well diffusion assay

Bacterial strain	Concentration of flower extracts tested (mg/mL)	DMSO final concentration (%)	Inhibition zone diameter (mm) of DMSO
E. coli	500	99.9	0
E. coli O157:H7	500	99.9	0
S. Typhi	500	99.9	0
S. dysenteriae	500	99.9	0
V. cholerae	500	99.9	0

B. MIC and MBC

Bacterial strain	Concentration of flower extracts tested (mg/mL)	DMSO final concentration (%)	DMSO concentration of MIC/ MBC (%)
E. coli	500	99.9	50/50
E. coli O157:H7	500	99.9	50/50
S. Typhi	500	99.9	50/50
S. dysenteriae	500	99.9	50/50
V. cholerae	500	99.9	50/50

C. Antibiofilm activity

Bacterial strain	Concentration of flower extracts tested (mg/mL)	DMSO final concentration (%)	Inhibition of biofilm by DMSO (%)
**Effects on Adherence Biofilm**			
E. coli	*C. ternatea* at 125 mg/mL	25	64.03±3.11
	M. ferrea at 62.5 mg/mL<br>*M. siamensis* at 62.5 mg/mL	12	2.11±0.75
E. coli O157:H7	*C. ternatea* at 125 mg/mL	25	50.69±3.35
	M. ferrea at 62.5 mg/mL<br>*M. siamensis* at 62.5 mg/mL	12	31.77±0.52

S. Typhi	*C. ternatea* at 125 mg/mL	25	39.37±1.83
	M. siamensis at 62.5 mg/mL	12	40.55±3.15
	M. ferrea at 31.25 mg/mL	6	16.75±3.00
S. dysenteriae	*C. ternatea* at 125 mg/mL	25	6.04±0.06
	M. ferrea at 31.25 mg/mL	6	0
	M. siamensis at 31.25 mg/mL		
V. cholerae	*C. ternatea* at 125 mg/mL	25	24.9±1.49
	M. siamensis at 62.5 mg/mL	12	0
	M. ferrea at 31.25 mg/mL	6	0
**Effects on Established Biofilm**			
E. coli	*C. ternatea* at 125 mg/mL	25	7.75±1.10
	M. ferrea at 62.5 mg/mL	12	8.77±0.02
	M. siamensis at 62.5 mg/mL		
E. coli O157:H7	*C. ternatea* at 125 mg/mL	25	50.48±1.90
	M. ferrea at 62.5 mg/mL	12	34.29±1.58
	M. siamensis at 62.5 mg/mL		
S. Typhi	*C. ternatea* at 125 mg/mL	25	14.95±0.67
	M. siamensis at 62.5 mg/mL	12	7.47±0.62
	M. ferrea at 31.25 mg/mL	6	5.75±1.18
S. dysenteriae	*C. ternatea* at 125 mg/mL	25	0
	M. ferrea at 31.25 mg/mL	6	0
	M. siamensis at 31.25 mg/mL		
V. cholerae	*C. ternatea* at 125 mg/mL	25	27.19±1.79
	M. siamensis at 62.5 mg/mL	12	22.66±1.71
	M. ferrea at 31.25 mg/mL	6	13.42±0.91

D. Antibacterial Adhesion Activity

Bacterial strain	Concentration of flower extracts tested (mg/mL)	DMSO final concentration (%)	Inhibition of bacterial adhesion by DMSO (%)
All tested bacteria	*C. ternatea* at 0.63 mg/mL	0.126	0
	M. ferrea at 0.08 mg/mL	0.016	0
	M. siamensis at 0.0024 mg/mL	0.00048	0

Table S6. IC_{50} values and selectivity index (SI) of flower extracts.

Flower Extract	IC_{50} (mg/mL)	MIC (mg/mL)	Selectivity Index (SI)
Mesua ferrea	2.37	62.5	0.038
Mammea siamensis	6.44	62.5	0.103
Clitoria ternatea	2.82	125	0.0226

IC_{50} = Concentration of the extract that reduces cell viability by 50%, determined by cell viability assay.

MIC = Minimum inhibitory concentration of the extract against pathogenic bacteria.

Selectivity Index (SI) = IC_{50}/MIC; values greater than 1 indicate higher selectivity toward bacteria than human cells.

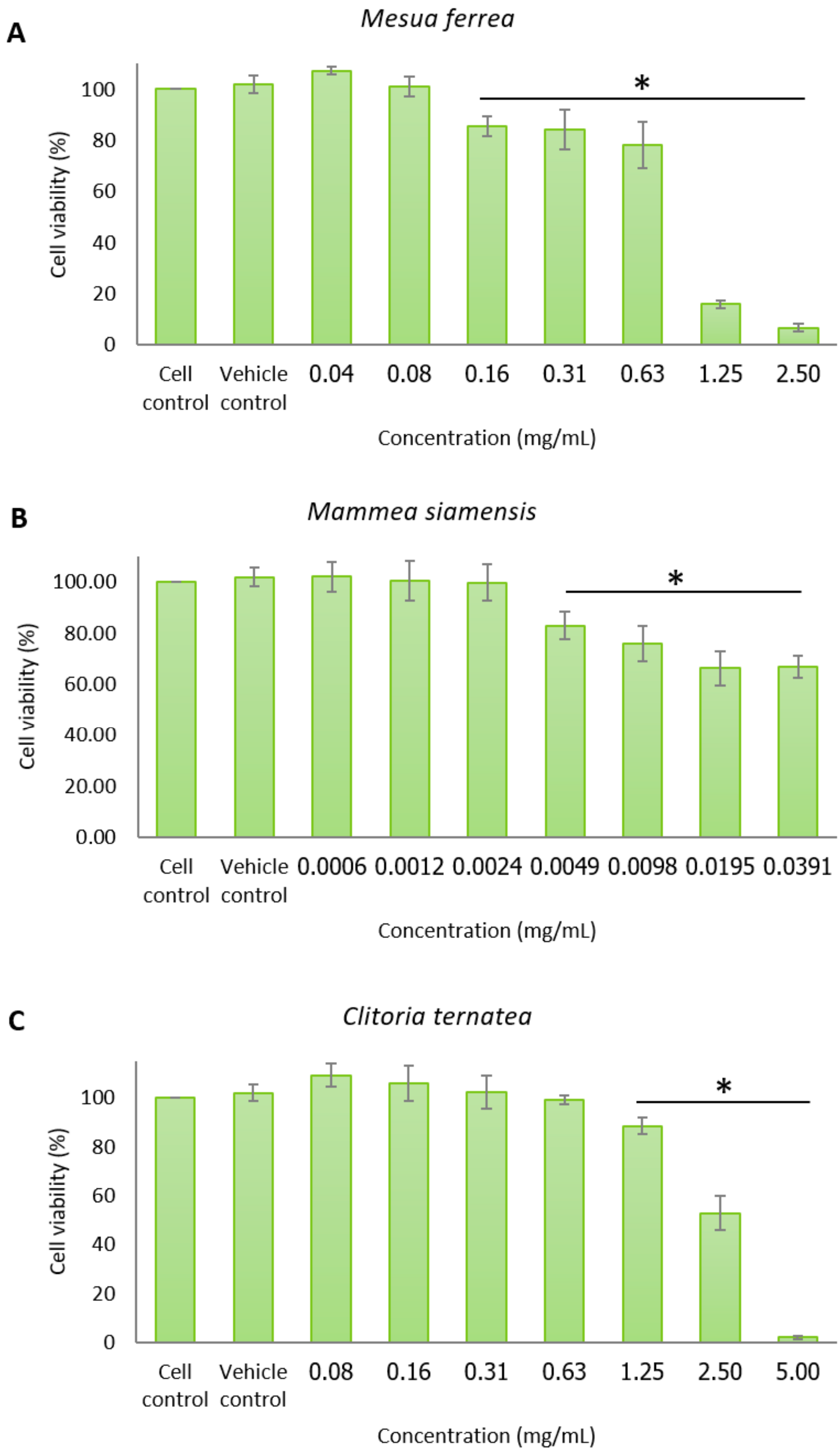

Figure S1. Effect of flower extracts on the viability of Caco-2 cells.

Vehicle control: 1% DMSO, equivalent to the flower extracts concentration of 5 mg/mL.

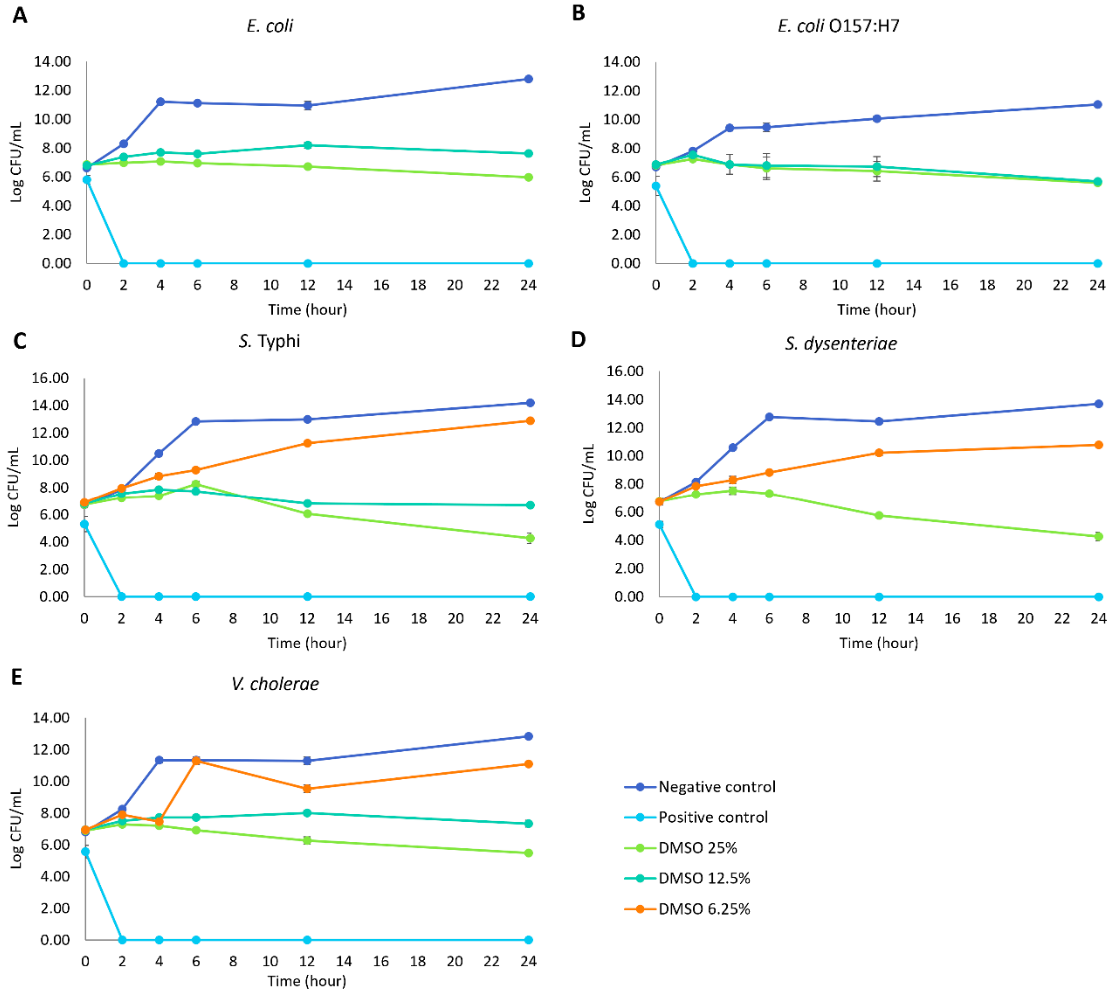

Figure S2. Effect of DMSO on time kill assay of flower extracts.
